# Characteristics Associated with Citation Rate of the Medical Literature

**DOI:** 10.1371/journal.pone.0000403

**Published:** 2007-05-02

**Authors:** Abhaya V. Kulkarni, Jason W. Busse, Iffat Shams

**Affiliations:** 1 Division of Population Health Sciences, Hospital for Sick Children, Toronto, Ontario, Canada; 2 Department of Clinical Epidemiology and Biostatistics, McMaster University, Hamilton, Ontario, Canada; University of California, San Francisco, United States of America

## Abstract

**Background:**

The citation rate for articles is viewed as a measure of their importance and impact; however, little is known about what features of articles are associated with higher citation rate.

**Methodology/Principal Findings:**

We conducted a cohort study of all original articles, regardless of study methodology, published in the *Lancet, JAMA*, and *New England Journal of Medicine*, from October 1, 1999 to March 31, 2000. We identified 328 articles. Two blinded, independent reviewers extracted, in duplicate, nine variables from each article, which were analyzed in both univariable and multivariable linear least-squares regression models for their association with the annual rate of citations received by the article since publication. A two-way interaction between industry funding and an industry-favoring result was tested and found to be significant (p = 0.02). In our adjusted analysis, the presence of industry funding and an industry-favoring result was associated with an increase in annual citation rate of 25.7 (95% confidence interval, 8.5 to 42.8) compared to the absence of both industry funding and industry-favoring results. Higher annual rates of citation were also associated with articles dealing with cardiovascular medicine (13.3 more; 95% confidence interval, 3.9 to 22.3) and oncology (12.6 more; 95% confidence interval, 1.2 to 24.0), articles with group authorship (11.1 more; 95% confidence interval, 2.7 to 19.5), larger sample size and journal of publication.

**Conclusions/Significance:**

Large trials, with group authorship, industry-funded, with industry-favoring results, in oncology or cardiology were associated with greater subsequent citations.

## Introduction

The dissemination of important research findings through the medical community begins with publication in peer-reviewed journals, but is continued through citation of the original work in subsequent publications. The number of citations received by an article is viewed as a marker for the importance of the original research and is reflected in the impact factor of journals in which the original paper was published. The impact factor is calculated as the mean number of citations received in a year for all articles published in the journal in the previous 2 years [1].

Although reference bias in the medical literature has been well established, the tendency to over-represent studies with positive findings [2]–[5], limited work has been done to determine what variables affect the number of citations an original paper will receive [6]–[8]. We therefore undertook a study to determine what factors were associated with an increased rate of citation using a cohort of articles published in leading medical journals. In particular, we examined whether certain variables that have been empirically linked to study quality or bias had a positive or negative impact on subsequent citation rate.

## Methods

### Selection of Journals and Articles

Our cohort of articles assembled included all original research papers published in the 6 month period between October 1, 1999 and March 31, 2000 appearing in the three general medical journals with the highest impact factors according to the Institute of Scientific Information's Journal Citation Report (ISI-JCR): the *New England Journal of Medicine* (NEJM), the *Journal of the American Medical Association* (JAMA), and the *Lancet*. We included all articles under the following table of content headings, regardless of study methodology: “Original Articles” in *NEJM*, “Original Contributions” in *JAMA*, and “Original Research–Articles” in *Lancet*. We excluded all other articles, including editorials, review articles, special articles, case reports, and research letters.

### Data Extraction

Two reviewers (AVK and JWB) trained in health research methodology extracted data independently and in duplicate, for the following variables: 1) the journal in which the article appeared (*NEJM*, *JAMA*, or *Lancet*) and the month of publication; 2) study design (randomized trial, prospective observational study, retrospective study, survey study, or meta-analysis); 3) clinical category of the article, defined as the medical subspecialty to which the main conclusion of the article was most applicable: anesthesiology, cardiovascular, dermatology, endocrinology, gastroenterology, general medicine, infectious disease, musculoskeletal, nephrology, neurology, obstetrics/gynecology, oncology, ophthalmology, pediatrics, psychiatry, or respirology; 4) whether the author by-line for the article included group authorship; 5) country in which the research was performed (defined as the country or countries in which research participants were recruited or, for research which did not use research participants, e.g., meta-analyses, the country of the corresponding author); 6) sample size of the study (in cases of meta-analysis, the sample size was taken as the total number of patients in all analyzed studies); 7) if an industry-affiliated drug or device was under investigation and whether the results favored the intervention or not; 8) declared industry funding; and 9) if the study had been reported in the lay media. Reviewers resolved discrepancies by discussion.

Industry was defined as for-profit companies and excluded all government agencies and non-profit private agencies. Industry funding was considered present if there was any acknowledgement of direct industry support for the research study (including direct funding of the study or supplying of drugs or medical devices). This did not include author-declared conflicts arising from having received individual consultant fees, for example.

In cases where studies explored the efficacy of an industry-affiliated device or drug two reviewers (AVK and JWB) independently evaluated whether the results would be considered favorable to industry. There is no standardized definition of positive results [9], and we considered study results favorable to industry if study findings suggested beneficial health effects or absence of expected adverse health effects with regards to the intervention under study. Disagreement was resolved through discussion. To explore the reliability of assessing industry-favouring status prior to data extraction, the same two reviewers independently evaluated 20 randomly selected studies from our cohort using a computer-based random number generator and found very good inter-observer reliability (kappa = 0.80).

To inform public interest in each study we searched the Associated Press health news wire during the 6-month period following publication of each article to determine if the study had been reported by the lay media. All data were extracted prior to determination of our primary outcome measure-the number of citations received.

### Outcome Measure Assessment

The primary outcome measure (annual rate of citation) was defined as the number of citations received per year since publication. Approximately five years (ranging from 57 months to 63 months) after we assembled our cohort, we conducted a citation search using the Institute of Scientific Information's (ISI) electronic version of *Science Citation Index* (http://isiknowledge.com) for each article, using a cited reference search, to determine the number of times the article had subsequently been cited in the medical literature. All citation searches were carried out in a single one week period in December 2004. A citation is counted by ISI if an article appears in a reference list in any of the approximately 8700 journals indexed by ISI. This would include reference lists associated with scientific papers, editorials, letters, or general interest articles. The initial query was performed by two of us independently (AVK and JWB) using the first author's name or group authorship name, journal title, and year of publication. If this query failed to yield any citations for an article, we conducted a search for the study title to limit misclassification of an article as having zero subsequent citations.

### Statistical Analysis

We performed all analyses using SPSS 13.0 statistical software (SPSS Inc., Chicago, IL). Amongst the 16 subgroups within the clinical category variable, only those with at least 20 articles each were retained as distinct subgroups for analysis; all other subgroups were combined into “others”. The country in which the research was performed was analyzed as either exclusively/partially in the United States or exclusively outside of the United States. Because of the highly skewed distribution of sample size (mean of 53310, but ranging from 1 to 3.3 million) we used a log10-transformation for this analysis. As funding source and study conclusions have been shown to be associated [10], so-called “sponsorship bias” [11], we decided, *a priori*, to test declared industry funding and industry-favoring results for interaction. We calculated the median and mean (with associated standard deviation [SD] or 95% CI) annual citation rate for all articles.

We used linear least-squares regression with the annual rate of citation as the dependent variable to explore associations. Each of the independent variables was initially tested in a univariable regression model. The F-test was used to calculate the level of significance and we included variables in our multivariable model if their level of significance was p<0.10 or they substantially altered the significance of another variable in the model. We used a step-forward method for entry into our multivariable analysis, in order from lowest p-value to highest. A variable was considered statistically significant if it had a p-value<0.05 in the final multivariable model. Multicollinearity was deemed concerning if the variance inflation factor for any independent variable was greater than 5 [12].

## Results

Our literature search generated 328 articles that were grouped into the following clinical categories: infectious disease (n = 62), cardiovascular (n = 57), oncology (n = 30), general medicine (n = 29), and obstetrics/gynecology (n = 25), leaving 125 articles assigned to “other”. Ninety-two (28.0%) studies were randomized and 68 (20.7%) were group authored (either exclusively or in addition to named individual authors). The majority of studies were performed either partly or exclusively in the United States (54.0%, 177 of 328). Eighty-two articles (25.0%) declared industry funding, of which approximately half (n = 42) reported industry-favoring results. Thirty-four studies reported industry-favoring results, but were not industry-funded. Ninety-seven articles (29.6%) had been reported by the Associated Press. (Table 1)

**Table 1:** Study Features Associated with Annual Citation Rate

Variable	Number of articles	Univariable Analysis (unstandardized regression coefficients (95% CI))	p-value	Multivariable Analysis (unstandardized regression coefficients (95% CI))	p-value
Industry funding			<0.001		0.002
-no	246	reference category		See interaction term *	
-yes	82	19.9 (12.2 to 27.5)			
Industry favoring result			<0.001		0.004
-no	252	reference category		See interaction term *	
-yes	76	19.4 (11.6 to 27.3)			
Industry funding & Industry favoring result (interaction term) *			n/a†		0.02
-no industry funding and no industry favoring result	212	n/a†		reference category	
-industry funding, but no industry favoring result	40	n/a†		3.9 (−6.1 to 14.0)	
-no industry funding, but industry favoring result	34	n/a†		2.5 (−8.2 to 13.2)	
-industry funding and industry favoring result	42	n/a†		25.7 (8.5 to 42.9)	
Clinical category			0.001		0.004
-other	125	reference category		reference category	
-cardiovascular	57	17.8 (8.1 to 27.5)		13.31 (3.9to 22.3)	
-general medicine	29	8.4 (−4.1 to 20.9)		9.1 (−3.2 to 21.5)	
-oncology	30	13.5 (1.2 to 25.8)		12.6 (1.2 to 24.0)	
-infectious disease	62	−0.7 (−10.1 to 8.7)		−2.9 (−11.7 to 5.8)	
-obstetrics & gynaecology	25	−7.7 (−21.0 to 5.6)		−6.5 (−19.0 to 5.9)	
Group authorship			<0.001		0.01
-no	260	reference category		reference category	
-yes	68	20.3 (12.2 to 28.5)		11.1 (2.7 to 19.5)	
Journal of publication			<0.001		0.002
-NEJM	102	reference category		reference category	
-JAMA	100	−8.6 (−17.2 to 0.0)		−9.7 (−18.0 to −1.5)	
-Lancet	126	−16.3 (−24.4 to −8.2)		−13.1 (−20.1 to −5.6)	
Sample size (log10 transformed)		3.1 (0.2 to 6.0)	0.04	2.8 (0.1 to 5.7)	0.04
News media coverage of article			<0.001		n/a†
-no	231	reference category		n/a†	
-yes	97	13.5 (6.1 to 20.9)		n/a†	
Location of study			0.001		n/a†
-not in United States	151	reference category		n/a†	
-partly/exclusive in United States	177	11.9 (5.2 to 18.7)		n/a†	
Study design			0.01		n/a†
-randomized	92	13.4 (−2.1 to 28.8)		n/a†	
-prospective	108	1.2 (−14.1 to 16.4)		n/a†	
-retrospective	92	−2.4 (−17.8 to 13.0)		n/a†	
-meta-analysis	15	2.8 (−18.4 to 23.9)		n/a†	
-survey	19	reference category		n/a†	
Month of publication (from 1 = October 1999 to 6 = March 2000)		0.7 (−1.3 to 2.7)	0.50		n/a†

95% CI = 95% confidence interval

† n/a = not applicable (variable was not included in the model)

* Since a significant interaction term between these two variables was included in the final multivariable model, their effects are dependent on each other and are presented together.

Our 328 eligible articles were cited a total of 38,381 times and the annual rate of citation ranged from 1.0 to 392.9 (median 14.1; mean 23.8, SD = 31.6).

Univariable regression models using annual rate of citation as the dependent variable yielded p-values<0.10 for all independent variables, except month of publication (p = 0.50).(Table 1) The variance inflation factors of all independent variables were less than 2.1, suggesting that multicollinearity was not a concern. Graphical examination of residuals against predicted values did not suggest a violation of the linearity assumption for the independent variables.

The following variables were retained in our multivariable regression model: industry funding, industry-favoring result, clinical category of article, group authorship, journal of publication, and sample size. (Table 1)

Based on our *a priori* hypothesis, a two-way interaction between industry funding and industry-favoring result was tested and found to be significant (p = 0.02). Therefore, if a study was industry funded, an industry-favoring result was associated with a significantly higher annual citation rate (an increase of 21.7; 95% CI = 9.2 to 34.3). However, if a study was not industry funded, a favorable result was not associated with a significant difference in annual citation rate (an increase of 2.5; 95% CI = −8.2 to 13.2). The unstandardized regression coefficients presented in Table 1 represent the difference in the annual citation rate between the subgroup and the reference category. Our model explained approximately 20% of the variance (adjusted R^2^ = 0.20) in annual citation rates of our cohort.

## Discussion

### Findings

Our analysis of a consecutive cohort of 328 original articles published in leading general medical journals found that declared industry funding with industry-favoring results, articles reporting data related to oncology or cardiovascular medicine, group authorship, higher impact journal of publication, and larger sample size were associated with higher rates of subsequent annual citation. Studies that declared industry funding and reported industry-favoring results were associated with the largest increase in annual citation rate.

### Limitations and Strengths

Our review has potential limitations. Despite our aggressive search strategy it is possible that some citations were missed, and the difficulty in accurately retrieving citations of group-authored articles, in particular, has been documented [13], [14]. However, this is likely to be only a relatively small proportion (only 10 articles in our sample were exclusively group-authored) which would be unlikely to substantially alter our main results. Further, we found group authorship was associated with greater citations which provides additional assurance that our search strategy was successful.

We did not assess self-citation, which has been associated with increased frequency of subsequent citation [15], [16]. As well, we assumed all subsequent citations to be quantitatively equal and we did not assess the context in which the citation appeared. For example, there may be differences between studies that are cited in a positive fashion versus those that are cited in a critical or negative fashion.

Despite including many potentially relevant independent variables, our final model only accounted for a moderate amount of the variability in the citations received (adjusted R^2^ = 0.20). Our model, however, was able to provide more explanation of the variance in citation frequency than the previous model by Callaham et al. (*pseudo*-R^2^ = 0.14) [6] and this is likely due to our inclusion of declared industry funding and industry-favoring results as variables. In fact, when these variables are removed from our model, the adjusted R^2^ falls to 0.15.

Our multivariable analysis highlights some of the limitations in the interpretation of the impact factor. For example, using our data, the difference in the annual citation rate between articles appearing in the highest and lowest impact journals in our sample (NEJM and Lancet) was 16.3, roughly in keeping with the 15.8 difference in their 2001 impact factors (29.1 and 13.3, respectively). However, the adjusted difference in annual citation rate was approximately 10.0 (95% CI = 1.7 to 18.3) (see Table 1), highlighting the fact the impact factor is attributable to more than just the journal of publication.

Our work has additional strengths. Our cohort of 328 articles is the result of a systematic search. Our data collection was comprehensive and careful, including independent judgment and abstraction of data at all stages conducted by methodological trained reviewers, and use of targeted, relevant analyses. Our results are not, however, generalizable to articles published in periodicals aside from the 3 high-impact general medical journals we reviewed.

### Implications

The rate of citation is used to calculate journal impact factors, which are viewed as a sign of journal importance and prestige. Subsequent citation and journal impact factor are commonly used as criteria for academic promotions within universities and the works of more accomplished researchers, including Nobel laureates, receive more citations than the works of other researchers [17]. Citation of articles is also an essential component of the dialogue of medical research–a dialogue which occurs largely within the pages of peer-reviewed journals. By re-iterating published research, citation serves to further the influence of their results.

In a review of emergency medicine papers, Callaham et al. found that the impact factor of the publishing journal was associated with the largest increase in citation rates [6]. Their study included a broader range of journal impact factors (ranging from 0.23 to 24.5) than our study, which was limited to only three very high impact factor journals (ranging from 13.3 to 29.1 in 2001). In our analysis, there was an association between journal and citation rate and this was in the expected direction, with articles in the higher impact journals having a higher rate of citation.

The impact factor of a journal has empirically been shown to be associated with article quality in some studies [18] but not in others [6]. In our adjusted analysis, larger sample size was associated with a higher citation rate while the design of the study was not. Some authors have described an association between citations and newsworthiness [6], [7]; however, the presence of an Associated Press news story (an indicator of newsworthiness and general public interest) did not demonstrate a significant enough association with citation rate to be included in our final multivariable model (5.5 more citations per year (95% CI = −2.2 to 13.2, p = 0.20) when added to the existing multivariable model).

The incidence of group authorship in the medical literature has steadily increased over the last two decades [19]. In our analysis, group-authored articles received approximately 11.1 more citations per year than articles with only individually named authors, a result consistent with previous findings by Dickersin et al. [13]. One could hypothesize that papers with group authorship are potentially larger studies, of higher methodological rigor, and of possible greater general interest. However, our multivariable analysis attempted to correct for such confounding variables. We did not study the effect of self-citation, which may account for up to 20% of subsequent citations [15]. It can be hypothesized that with group authorship (and, therefore, a greater number of authors) the potential impact of self-citation may be greater, thereby at least partly accounting for the higher citation rate.

The potential bias associated with industry-sponsored research has been suggested in previous works that have found an association between industry funding and the reporting of favorable results [20]–[22] and lower methodological quality [18]. Friedberg et al. found that pharmaceutical company sponsorship of economic analyses was associated with reduced likelihood of reporting unfavorable results [23]. Djulbegovic et al. reported that industry-funded trials more often compared innovative treatments to either a placebo arm or no therapy, resulting in a higher proportion of such studies favoring the new intervention [24]. This type of research has generally concentrated on examining the association between industry funding and study results. However, the next step in the dissemination of results is through their subsequent citation, and Patsopoulos et al. have recently shown that the proportion of most frequently cited articles funded by industry has been increasing [25].

After controlling for a number of other independent variables our analysis found that studies with declared industry funding received approximately 22 more citations per year only if their results were industry-favoring. The influence of 22 additional citations per year certainly appears to be substantial when put in context to the impact factors of the most cited journals in general medicine (which range from 10.4 to 44.0 for the top five journals in 2005). Therefore, the added influence appears to be the quantitative equivalent of having an extra publication in a high-impact journal. These extra citations may have the effect of amplifying the results of these studies in the medical literature.

### Conclusions

In our analysis, large trials, with group authorship, industry-funded, with industry-favoring results, in oncology or cardiology were associated with greater subsequent citations. Declared industry funding with industry-favoring results was associated with the largest increase in annual citation rate. The medical community should be aware of the potential for these studies and their results to have greater impact in the subsequent medical literature.
